# Letter to the Editor: ‘Long-term quality of life between duodenum-preserving pancreatic head resection and pancreatoduodenectomy: a systematic review and meta-analysis’

**DOI:** 10.1097/JS9.0000000000001417

**Published:** 2024-04-19

**Authors:** Yong-Hui Sun, Nan Ding, Hai Lin, Kun Cheng, Hao Ding, Qi-Long Chen

**Affiliations:** a Digestion and Vascular Center, Department of Pancreas Surgery, The First Affiliated Hospital of Xinjiang Medical University; b Department of Pharmacy, The First Affiliated Hospital of Xinjiang Medical University, Urumqi; c Xinjiang Key Laboratory of Medical Animal Model Research, Urumqi; d Xinjiang Key Laboratory of Clinical Drug Research, Urumqi, People’s Republic of China

*Dear Editor,*

We read with great interest the article by Taoyuan *et al*.^1^ ‘Long-term quality of life between duodenum-preserving pancreatic head resection and pancreatoduodenectomy: a systematic review and meta-analysis’.

This study delved into the long-term differences in quality of life (QOL) and overall survival (OS) following duodenum-preserving pancreatic head resection (DPPHR) and pancreatoduodenectomy (PD), offering valuable insights for the selection of surgical approaches in pancreatic disease and contributing important evidence-based medicine for clinical decision-making in the real world. Notably, the research revealed that DPPHR was more beneficial than PD in enhancing OS, undoubtedly providing significant guidance for clinicians dealing with chronic pancreatitis. The patient cohort included in this study primarily consisted of those with chronic pancreatitis, with some undergoing the Frey, Berne, or Beger and others PD as the control group. Based on our center’s limited experience, patients selected for the Frey procedure typically have pancreatic duct stones, while those with mass-forming pancreatitis or non-extractable stones in the head of the pancreas are chosen for Beger or PD^2^. In cases opting for PD, the pancreas is often significantly fibrotic due to inflammation^3^, with a harder texture, making Beger’s procedure more challenging and necessitating the choice of PD. Consequently, patients in the PD group may exhibit more severe pancreatic fibrosis^4^, suggesting potentially diminished endocrine and exocrine functions compared to those undergoing Beger or Frey procedures, a factor that may impact OS and warrants further research for clarification.

Furthermore, patients in the DPPHR group retain the integrity of the digestive tract, especially the functionality of the duodenum^5^. Therefore, it is of interest whether there is a difference in trace elements between the two groups that could affect OS, an area yet to be explored by research. The difference in trace elements following DPPHR and PD and their potential impact are issues currently under investigation at our center, and we look forward to reporting our findings and eagerly anticipate collaborative research with other domestic and international centers to obtain more reliable results.

Additionally, while no significant differences were reported in global QOL scores, DPPHR showed advantages in reducing long-term symptoms such as fatigue, nausea, vomiting, appetite loss, insomnia, and diarrhea. Even minor symptoms can significantly impact daily living, and their improvement might substantially affect the overall QOL. It raises the question of whether more sensitive tools or assessment methods could be utilized to detect these subtle but important changes in QOL.

Moreover, long-term complications after PD, such as anastomotic site stones and reflux cholangitis, which might necessitate further surgery, can affect the patient’s physiological and psychological well-being as well as their QOL^2^. These long-term post-PD complications are worthy of attention in clinical decision-making, and their impact should be considered in future studies.

In this study, the authors noted that DPPHR showed superior perioperative outcomes, which may be attributable to the inclusion of modified procedures such as Frey and Berne. According to the current statistics from our center on Beger-related outcomes, the incidence of complications following Beger’s procedure is significantly higher compared to PD. The most common postoperative complications at our center following the Beger procedure are pancreatic fistula (44.90%), biliary fistula (38.78%), and delayed gastric emptying (28.57%). There was one postoperative death due to myocardial infarction, with no other cases of mortality recorded. We believe that the rate of postoperative complications associated with Beger’s procedure is notably higher than that with PD. Future studies should differentiate between Beger and other modified procedures such as Frey or Berne to obtain more precise data that can guide clinical practice.

Finally, considering that patient heterogeneity may influence treatment outcomes, I wonder if further stratified analyses might be possible, such as those based on specific pathology, pancreatic function status, or preoperative QOL gradings. Such stratification could help identify which patient cohorts could benefit more from DPPHR as opposed to PD.

I am grateful to Professor Yin Taoyuan and his team for their contributions to this field and look forward to further dialogue and exchange.

## Ethical approval

No ethical approval was required for this letter.

## Consent

Not applicable.

## Source of funding

No sources of funding were received.

## Author contribution

Y.-H.S.: wrote the manuscript; N.D., H.L., K.C., and H.D.: updated the manuscript; Q.-L.C.: reviewed and edited the manuscript. All authors have approved the final draft and are responsible for the manuscript.

## Conflicts of interest disclosure

The authors declares conflicts of interest.

## Research registration unique identifying number (UIN)

1. Name of the registry: not applicable.
2. Unique identifying number or registration ID: not applicable.
3. Hyperlink to your specific registration (must be publicly accessible and will be checked): not applicable.

## Guarantor

All authors.

## Data availability statement

Data not available/not applicable.

## Provenance and peer review

Not commissioned, internally reviewed.
